# Endoscopic third ventriculostomy for hydrocephalus in a patient with Klippel–Feil syndrome: a case report

**DOI:** 10.1002/ccr3.1063

**Published:** 2017-07-06

**Authors:** Tomohisa Ishida, Takashi Inoue, Miki Fujimura, Yoshiteru Shimoda, Masayuki Ezura, Hiroshi Uenohara, Teiji Tominaga

**Affiliations:** ^1^ Department of Neurosurgery Sendai Medical Center Sendai Miyagi Japan; ^2^ Department of Neurosurgery Tohoku University Graduate School of Medicine Sendai Miyagi Japan

**Keywords:** Endoscopic third ventriculostomy, hydrocephalus, Klippel–Feil syndrome

## Abstract

A patient with Klippel–Feil syndrome presented with hydrocephalus secondary to intraventricular hemorrhage. Fusion of the cervical vertebrae may have impeded cerebrospinal fluid flow. Change in the properties of cerebrospinal fluid flow after hemorrhage may have induced noncommunicating hydrocephalus. Endoscopic third ventriculostomy was effective for the treatment of hydrocephalus associated with Klippel–Feil syndrome.

## Introduction

Klippel–Feil syndrome was first described in a patient with a short neck, low posterior hairline, and limited range of motion of the neck caused by a congenital segmentation defect of the cervical spine 1. Klippel–Feil syndrome is characterized by malformation of the craniocervical skeleton, but numerous other abnormalities of other organ systems may also be present. Neurological defects are the most common anomalies associated with this syndrome, exclusive of hearing and visual disorders, and include mirror movement, mental retardation, and paralysis and palsy 2. Hydrocephalus may also occur and is usually treated with ventriculoperitoneal shunt 3.

We treated a patient with Klippel–Feil syndrome suffering from hydrocephalus, which was improved after endoscopic third ventriculostomy (ETV). We describe this case and discuss the therapeutic advantage of ETV in patients with Klippel–Feil syndrome.

## Case Report

**Figure 1:**
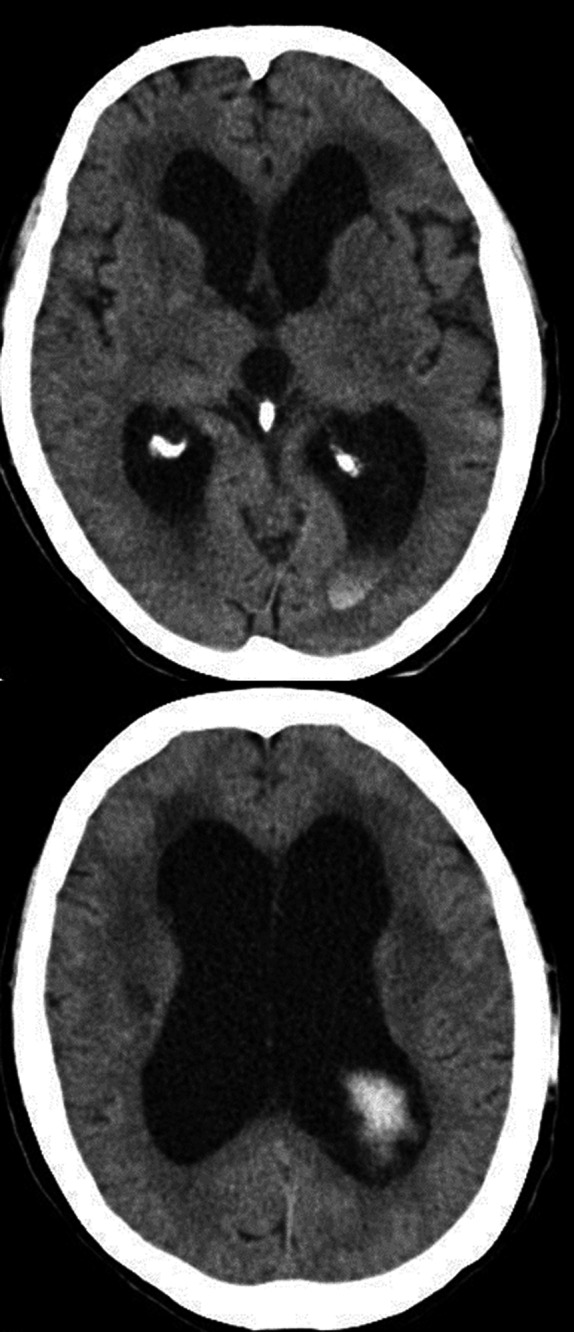
Computed tomography scan on admission showing ventricular enlargement and intraventricular hemorrhage.

**Figure 2:**
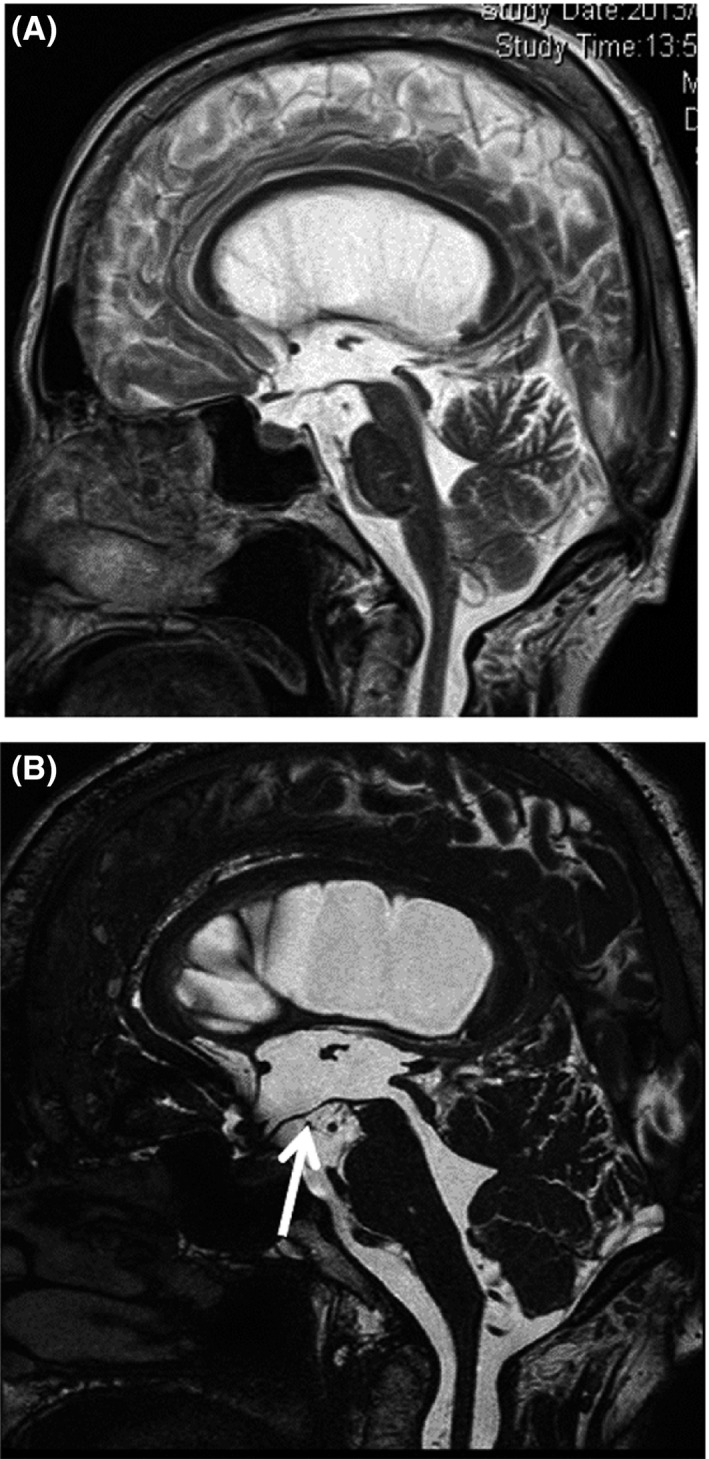
(A) Sagittal T2‐weighted magnetic resonance (MR) image 2 years before admission showing enlargement of the lateral, third, and fourth ventricles. (B) Sagittal T2‐weighted MR image on admission showing slight ballooning of the third ventricle floor (arrow), but no obvious obstruction.

**Figure 3:**
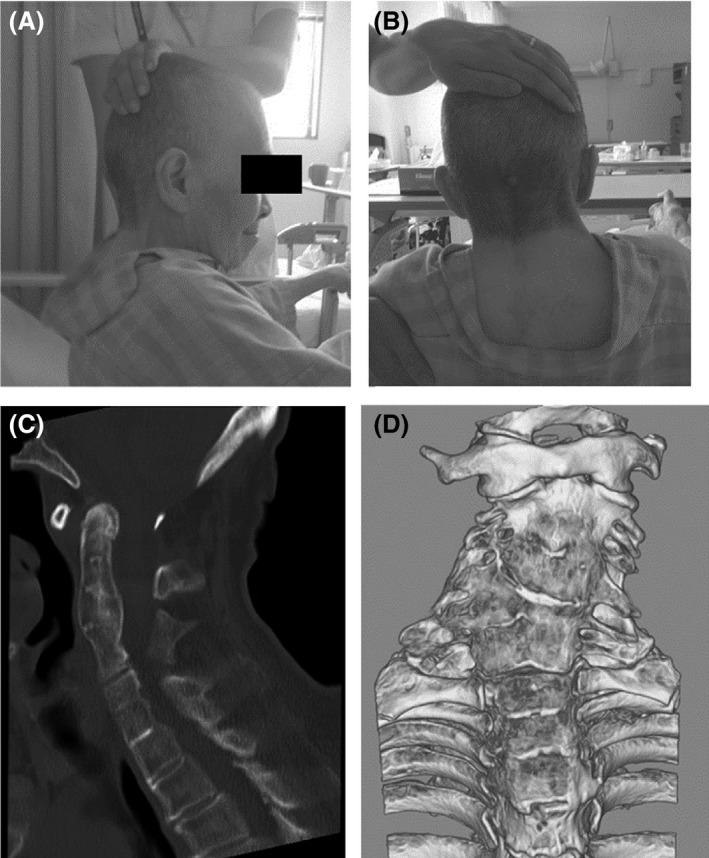
(A, B) Note short neck and low posterior hairline. (C, D) Lateral and posterior views of the cervical spine demonstrating fusion of the cervical vertebrae at C2–4 and C5–7.

A 75‐year‐old woman was referred to our hospital with intraventricular hemorrhage (Fig. 1) caused by hypertensive choroid plexus hemorrhage. She had a history of hearing loss from childhood. Cranial magnetic resonance (MR) imaging at age 73 years for screening of dementia demonstrated ventricular enlargement (Fig. 2A). On admission, she had mild conscious disturbance (Glasgow Coma Scale [GCS] 12: *E* = 4, *V* = 3, *M* = 5), gait disorder, and difficulty in feeding herself. She had short neck and low posterior hairline (Fig. 3A,B).

## Imaging

Radiography showed fusion of the cervical vertebrae at C2–4 and C5–7 (Fig. 3C,D). Cranial computed tomography and MR imaging demonstrated enlargement of the lateral, third, and fourth ventricles, and slight ballooning of the floor of the third ventricle. No indications of mass lesion were detected (Fig. 1B). The posterior cranial fossa volume measured by MR imaging was 145.4 mL. Based on these findings, the diagnosis was obstructive hydrocephalus associated with Klippel–Feil syndrome, with the suspected obstruction located at the outlet of the fourth ventricle.

## Treatment

**Figure 4:**
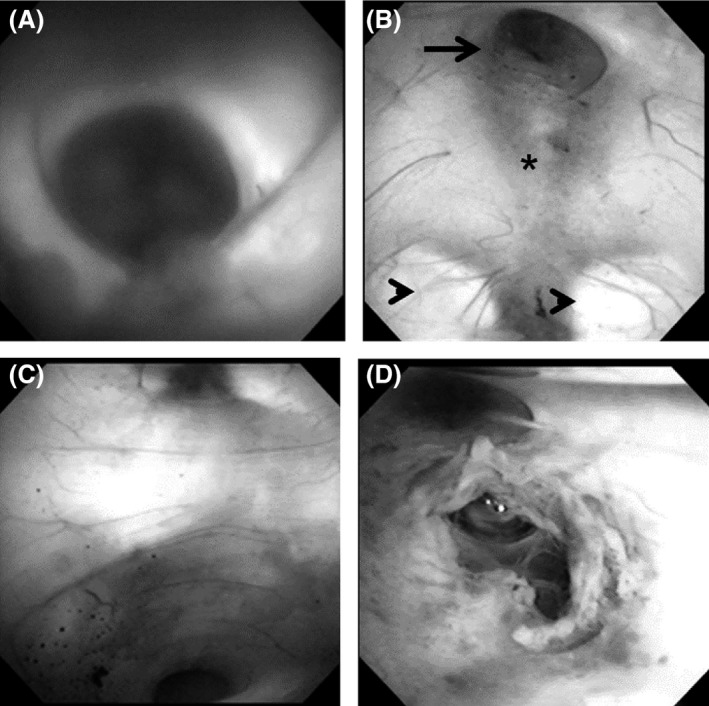
(A) Intra‐operative endoscopic view showing the enlarged foramen of Monro. (B) Intra‐operative endoscopic view of the third ventricle floor showing the landmark structures: infundibular recess (arrow), mammillary bodies (arrowhead), and tuber cinereum (asterisk). (C) Intra‐operative endoscopic view showing the funnel‐shaped enlarged aqueduct (up‐pointing triangle). (D) After fenestration of the third ventricle floor.

Endoscopic third ventriculostomy was performed for the hydrocephalus. An intraventricular videoscope was introduced into the left anterior horn of the lateral ventricle. The septum pellucidum showed thinning, and the foramen of Monro was enlarged (Fig. 4A). The third ventricle floor was approached via the foramen of Monro. The infundibular recess, mammillary bodies, and tuber cinereum were identified on the third ventricular floor (Fig. 4B). The aqueduct showed funnel‐shaped enlargement (Fig. 4C). Perforation was made just posterior to the infundibular recess and tuber cinereum, and the fenestration was widened using a balloon catheter. Patency of the cerebrospinal fluid (CSF) pathway was confirmed by checking the to‐and‐fro movement of the CSF (Fig. 4D). No surgical complication occurred during hospitalization. Her GCS score was improved to 14 (*E* = 4, *V* = 4, *M* = 6). She could feed herself and was transferred to another hospital for ambulatory rehabilitation.

## Discussion

Hydrocephalus is observed in about 2% of patients with Klippel–Feil syndrome 2. Several causes of hydrocephalus are known, but all are obstructive mechanisms. Chiari malformation and basilar impression may be present in patients with Klippel–Feil syndrome, which can result in hydrocephalus caused by obstruction of the egress of CSF from the fourth ventricle to the subarachnoid space 3.

In the present case, no evidence of Chiari malformation, basilar impression, or other abnormalities that could cause obstruction of CSF flow was found. The normal posterior cranial fossa volume is reported to be 165.6 ± 19.4 mL. The volume in the present case (145.4 mL) was smaller than this reported value 4. Such a smaller posterior fossa interferes with CSF circulation 5. In our patient, no obstruction was found throughout the ventricular system, but the floor of the third ventricle showed ballooning, which had not been observed by the previous imaging at 73 years old. We considered that the pressure in the third ventricle had increased. Treatment with ETV was effective, and her clinical symptoms improved after ETV. Therefore, the final diagnosis was noncommunicating hydrocephalus. In her case, Klippel–Feil syndrome may have impeded the CSF flow for a long time, and the change in CSF concentration caused by intraventricular hemorrhage had resulted in noncommunicating hydrocephalus.

In the present case, we considered that the functional obstruction or stenosis was located at the egress of the fourth ventricle as a result of fusion of the vertebrae, although we could not identify any obvious obstruction point. The selection of ETV could avoid shunt surgery, which carries the risk of infection. Therefore, we planned to perform ETV first, and then shunt surgery if the symptoms did not improve. We speculate that the ventricular enlargement had existed for a long time, caused by the functional obstruction associated with Klippel–Feil syndrome. Subsequently, the hydrocephalus developed because of increased CSF viscosity due to intraventricular hemorrhage.

Ventriculoperitoneal shunt was previously recommended for such cases 3. Our present case suggests that ETV may also be effective for the treatment of hydrocephalus associated with Klippel–Feil syndrome.

## Conclusion

Craniocervical deformation can impede CSF flow. Noncommunicating hydrocephalus can occur without obvious obstruction in patients with Klippel–Feil syndrome. ETV may be effective for the treatment of hydrocephalus associated with Klippel–Feil syndrome.

## Conflict of Interest

None declared.

## Authorship

TI: designed the study, and wrote the initial draft of the manuscript. TI: contributed to analysis and interpretation of data, and assisted in the preparation of the manuscript. All other authors: have contributed to data collection and interpretation, and critically reviewed the manuscript. All authors: approved the final version of the manuscript, and agreed to be accountable for all aspects of the work in ensuring that questions related to the accuracy or integrity of any part of the work are appropriately investigated and resolved.
